# Determining the Balance Between Drug Efficacy and Safety by the Network and Biological System Profile of Its Therapeutic Target

**DOI:** 10.3389/fphar.2018.01245

**Published:** 2018-10-31

**Authors:** Xiao xu Li, Jiayi Yin, Jing Tang, Yinghong Li, Qingxia Yang, Ziyu Xiao, Runyuan Zhang, Yunxia Wang, Jiajun Hong, Lin Tao, Weiwei Xue, Feng Zhu

**Affiliations:** ^1^College of Pharmaceutical Sciences, Zhejiang University, Hangzhou, China; ^2^School of Pharmaceutical Sciences and Collaborative Innovation Center for Brain Science, Chongqing University, Chongqing, China; ^3^Key Laboratory of Elemene Class Anti-cancer Chinese Medicine of Zhejiang Province, School of Medicine, Hangzhou Normal University, Hangzhou, China

**Keywords:** drug efficacy-safety balance, therapeutic index, artificial intelligence, protein-protein interaction network, biological system profile

## Abstract

One of the most challenging puzzles in drug discovery is the identification and characterization of candidate drug of well-balanced profile between efficacy and safety. So far, extensive efforts have been made to evaluate this balance by estimating the quantitative structure–therapeutic relationship and exploring target profile of adverse drug reaction. Particularly, the therapeutic index (TI) has emerged as a key indicator illustrating this delicate balance, and a clinically successful agent requires a sufficient TI suitable for it corresponding indication. However, the TI information are largely unknown for most drugs, and the mechanism underlying the drugs with narrow TI (NTI drugs) is still elusive. In this study, the collective effects of human protein–protein interaction (PPI) network and biological system profile on the drugs' efficacy–safety balance were systematically evaluated. First, a comprehensive literature review of the FDA approved drugs confirmed their NTI status. Second, a popular feature selection algorithm based on *artificial intelligence (AI)* was adopted to identify key factors differencing the target mechanism between NTI and non-NTI drugs. Finally, this work revealed that the targets of NTI drugs were highly centralized and connected in human PPI network, and the number of similarity proteins and affiliated signaling pathways of the corresponding targets was much higher than those of non-NTI drugs. These findings together with the newly discovered features or feature groups clarified the key factors indicating drug's narrow TI, and could thus provide a novel direction for determining the delicate drug efficacy-safety balance.

## Introduction

One of the most challenging puzzles in drug discovery is the identification and characterization of candidate drugs of well-balanced profile between efficacy and safety (Muller and Milton, 2012; Li et al., 2018; Xue et al., 2018b). In other words, apart from extensive effort made to optimize drug affinity and selectivity (Wang et al., 2017a; Zheng et al., 2017), considerable investments should be devoted to detect adverse drug reactions (Huang et al., 2018) and reveal drug likeness (Benet et al., 2016; Yang et al., 2018). So far, the identification of drug toxicities in preclinical or clinical developments has been accelerated by a variety of technological advances (Badders et al., 2018) including biomarker-guided safety assessment (Muller and Dieterle, 2009; Rzepecki et al., 2018), OMICs techniques (Iloro et al., 2013; Fu J. et al., 2018), breakthrough in computing capacity and bioinformatics method (Zhu et al., 2011; Tao et al., 2015; Chen et al., 2016), and so on. To measure the level of correlation between drug maximum efficacy and confined safety in given disorder, the therapeutic index (TI typically considered as the ratio of the highest non-toxic drug exposure to the exposure producing the desired efficacy) has emerged as a key indicator illustrating that delicate balance (Zaykov et al., 2016). The TI is essential for life-threatening diseases (such as cardiovascular and oncological disease) with limited treatment options (Zhu et al., 2008b; Kimmelman and Federico, 2017). Particularly, tiny variation in the dosage of drugs with narrow TI (NTI drugs, TI ≤3) may result in therapeutic failure or serious adverse drug reactions (Tao et al., 2014; Ewer and Ewer, 2015; Zheng et al., 2016), and is only acceptable for the treatment of life-threatening diseases (Yu et al., 2015). Therefore, successful therapeutic agents require sufficient TI (NNTI drugs, TI >3) suitable for it corresponding indication (Abernethy et al., 2011).

However, TI characterization is too complicated to be achieved for many drugs (Yu et al., 2015), and TI is highly susceptible to the subject variations of drug responses (Jiang et al., 2015; Yang et al., 2017). To enhance the determination and interpretation of TI, a variety of *in-silico* studies have been performed to reveal the mechanism underlying NTI drugs (Muller and Milton, 2012). In particular, the prediction models based on quantitative structure–activity (QSAR), structure–toxicity (QSTR), and structure–index (QSIR) relationship have been constructed to enable early assessment of TI (Zhu H. et al., 2008; Rodgers et al., 2010; Zhu et al., 2012a; Chen et al., 2016; Fu T. et al., 2018). These models are primarily constructed and exert their prediction capacity based on structures of the studied drugs, which thus demonstrate great limitations in coping with TI's vulnerability to the subject variation of drug responses (Jiang et al., 2015). Compared with the approaches based on drug structure, target-based approach turns out to be the one of enhanced effectiveness for characterizing confined toxicity behind the drug efficacy (Muller and Milton, 2012; Huang et al., 2018), since the population variation of drug target is capable of reflecting, to some extent, the subject variations of drug responses (Fujimoto et al., 2014; Jiang et al., 2015). But target-based method is sophisticated due to the involvement of target in complex protein–protein interaction (PPI) network (Rao et al., 2011; Li et al., 2016b; Xu et al., 2016; Wang et al., 2017b) and the necessity of considering target biological system profiles (Zhu F. et al., 2009; Xue et al., 2016).

So far, the PPI network properties (Ragusa et al., 2010; Guo et al., 2018) and biological system profiles (Zheng et al., 2006) have been adopted to analyze the drug likeness of candidate agents. On one hand, the target–protein interaction network has been constructed and the corresponding network features can be calculated for discovering the differential properties indicating disease status (Ragusa et al., 2010) and identifying candidate drug targets for a given indication (Guo et al., 2018; Xue et al., 2018a). On the other hand, the druggability of candidate target is found significantly determined by a variety of biological system profiles, which include the number of target affiliated signaling pathways (Yang et al., 2016), the number of similarity proteins outside target's protein family (Zheng et al., 2006), the number of human tissues distributed by the studied target (Zhu F. et al., 2009), and the differential level of target expression between patient and healthy individual (Ernst et al., 2017; Li et al., 2018). Since the underlying theories of network- and biological system-based approaches are distinct from each other (Guo et al., 2018; Li et al., 2018), it is essential to simultaneously consider these two types of properties for understanding drug likeness. However, these properties have not yet been collectively considered in TI-related studies, and the mechanism underlying drugs' narrow TI is still elusive.

In this study, a comprehensive analysis on the network features and biological system profiles of the primary therapeutic targets of all FDA approved drugs was conducted, and various features differentiating drugs of narrow TI (NTI drugs) from those of sufficient TI (NNTI drugs) were identified. First, due to the limited information of both NTI and NNTI drugs, a systematic literature review was conducted to collect the TI data for all approved drugs. Then, the primary therapeutic targets of these drugs were classified into four groups based on collected TI data. These four target groups include (a) targets of NTI drugs, (b) targets of both NTI and NNTI drugs, (c) targets of drugs without reported TI, and (d) targets of NNTI drugs. Third, a comparative analysis between target group (a) and (d) identified several key features able to differentiate two groups, and further study revealed three feature groups indicating the mechanisms underlying NTI drugs. In summary, these findings together with the newly discovered features or feature groups clarified key factors indicating drug's narrow TI, which gave a new direction for determining the delicate balance between drugs' maximum efficacy and confined safety.

## Materials and methods

### Systematic collection of drugs and their corresponding targets and TI data

The TI data of FDA approved drugs were obtained by four steps. First, FDA approved drugs were collected from the official website of FDA (Drugs@FDA), and their corresponding diseases were carefully confirmed. In total, 1,762 drugs were collected. Second, the primary therapeutic targets of these drugs were identified from the TTD database (https://db.idrblab.org/ttd/; Li et al., 2018), and 418 primary therapeutic targets of these 1,762 drugs were discovered (detail information was provided in the following paragraphs). Third, TI data of these drugs were systematically collected by a comprehensive literature review. Particularly, various keyword combinations were searched in PubMed and other academic resources, which included “drug name + therapeutic index,” “drug name + therapeutic window,” “drug name + critical dose,” “drug name + therapeutic ranges,” and “drug name + therapeutic ratio.” As a result, 161 NTI and 29 NNTI drugs confirmed by the clinical evaluations or experiments were identified, which aimed at 60 and 28 human targets, respectively. Supplementary Table S1 provided a full list of 161 NTI and 29 NNTI drugs together with their approved disease indication and corresponding targets. To the best of our knowledge, it is the first comprehensive literature review on the TI data of all drugs approved by FDA and Supplementary Table S1 provided the most completed information of the FDA approved drugs with available TI data. Moreover, the primary therapeutic targets of all FDA approved drugs were classified into four groups based on their TI: (a) 20 targets of NTI drugs, (b) 40 targets of both NTI and NNTI drugs, (c) 339 targets of drugs without reported TI, and (d) 19 targets of NNTI drugs. Moreover, among those drugs listed in Supplementary Table S1, four multi-target drugs were found with NTI data available, which included *regorafenib* (hepatocellular and colorectal cancer), *sorafenib* (renal cell and hepatocellular carcinoma), *sunitinib* (gastrointestinal cancer), and *vandetanib* (medullary thyroid cancer). All these drugs are multi-kinases inhibitors for the treatment of cancer.

### Identification of the primary therapeutic target(S) of FDA approved drugs

The primary therapeutic target of each FDA approved drug was strictly determined by considering (1) the experimentally determined potency of drugs against their primary target or targets (Zhu et al., 2010), (2) the observed potency or effects of drugs against disease models (cell lines, *ex-viv*o, *in-vivo* models) linking to their primary drug targets (Zhu et al., 2012b), and (3) the observed effect of target knockout, knockdown, transgenetic, RNA interference, antibody or antisense-treated *in vivo* models (Zhu et al., 2012b). Taking the confirmation of CDK4 as the primary therapeutic target of FDA approved Palbociclib as an example, it was determined by considering: (1) experimentally defined high potency (IC50 = 11 nM) of Palbociclib against CDK4 (Fry et al., 2004), (2) the clearly observed development of multiple tumors by a point mutation (R24C) in the first coding exon of locus encoding CDK4 in the mice models (Sotillo et al., 2001), and (3) Palbociclib-induced G1-G2 arrest and apoptosis in breast tumor cell lines (IC50 <400 nM) and tumor growth reduction in human breast tumor xenograft (Lapenna and Giordano, 2009). In conclusion, only the targets with complete target determination data (including all three types of information above) were defined as the primary therapeutic targets of the corresponding FDA approved drugs.

### Deriving the human PPI network properties for each studied target

The human protein–protein interaction (PPI) network analyzed here included 15,554 proteins and 642,304 PPIs, which was constructed using the data provided in STRING (Szklarczyk et al., 2015). In order to ensure the reliability of the analyzed data, only those PPIs with high confidence score (>0.95) were collected for the subsequent analyses (Ghosh et al., 2015; Wang S. et al., 2015). As a result, a sub-network with 8,509 proteins and 40,468 PPIs were generated and adopted for further analyses in this study. Moreover, the network properties for each studied target were generated by the PROFEAT (Zhang et al., 2017a) and the tool NetworkAnalyzer of Cytoscape (Shannon et al., 2003; Thomas and Bonchev, 2010).

In total, 32 network properties were calculated and adopted in subsequent analysis. These properties were popular for analyzing a complex biological network, which included: (1) *Average Closeness Centrality*: the average number of steps required to reach the studied node from any node in a network (Ma et al., 2016); (2) *Average Shortest Path Length*: the average length of shortest paths between the studied node and all other ones (Zhang et al., 2014); (3) *Betweenness Centrality*: the number of times the studied node serving as a linking bridge along shortest path between any two nodes (Zeidán-Chuliá et al., 2015); (4) *Bridging Centrality*: the product of the bridging coefficient and betweenness centrality (Hwang et al., 2008); (5) *Bridging Coefficient*: the extent of the studied node lying between any other densely connected nodes in the network (Paladugu et al., 2008); (6) *Closeness Centrality Sum*: the reciprocal of the sum of the shortest paths between the studied node and all other nodes in the network (Costenbader and ValenteFontanesi, 2003); (7) *Clustering Coefficient*: the number of the connected pairs between all neighbors of node (Watts and Strogatz, 1998); (8) *Current Flow Betweenness*: a centrality index measuring the level of information travels along all possible paths within network (Paladugu et al., 2008); (9) *Current Flow Closeness*: the variant of current flow betweenness (Zhang et al., 2017b); (10) *Degree*: the number of edges linked to a node (Braeuning, 2013); (11) *Degree Centrality*: the number of links incident upon a studied node (Batool and Niazi, 2014); (12) *Deviation*: the variation between sum of node distances and network unipolarity (Zhang et al., 2017a); (13) *Distance Deviation*: the absolute difference between nodes' distance sum and network's average distance (Rogelj et al., 2013); (14) *Distance Sum*: the sum of all shortest paths starting from the studied node (Bolser et al., 2003); (15) *Eccentric*: the absolute difference between nodes' eccentricities and network's average eccentricity (Zhang et al., 2017a); (16) *Eccentricity*: the maximum non-infinite shortest path length between the studied node and all other nodes in the network (Bolser et al., 2003); (17) *Eccentricity Centrality*: the largest geodesic distance between the node and any other node (Batool and Niazi, 2014); (18) *Eigenvector Centrality*: the sum of its neighbors' centrality values (Solá et al., 2013); (19) *Harmonic Closeness Centrality*: the sum of the reciprocals of the average shortest path lengths of each node in network (Zhang et al., 2017b); (20) *Interconnectivity*: a connectivity index indicating the quality of the studied nodes being connected together (Emig et al., 2013); (21) *Load Centrality*: the fraction of all the shortest paths that pass through the studied node (Kivimäki et al., 2016); (22) *Neighborhood Connectivity*: the average connectivity of all neighbors (Carson and Lu, 2015); (23) *Normalized Betweenness*: the fraction of network shortest paths that a given protein lies on (Paladugu et al., 2008); (24) *Number of Self Loops*: the number of edges starting and ending at the same node (Garlaschelli and Loffredo, 2004); (25) *Number of Triangles*: the number of triangles that include the studied node as a vertex (Rubinov and Sporns, 2010); (26) *Page Rank Centrality*: an adjustment of Katz by considering the diluted issue (Li et al., 2013); (27) *Radiality*: the level of reachability of a studied node via various shortest paths within the entire network (Koschützki and Schreiber, 2008); (28) *Residual Closeness Centrality*: the closeness measured by removing the studied node (Dangalchev, 2006); (29) *Scaled Degree*: the degree of a studied node relative to the most connected node within the same module (Sormani, 2012); (30) *Stress*: the number of shortest paths passing through a given node (Shannon et al., 2003); (31) *Topological Coefficient*: the extent to which a node in network shares interaction partners with other nodes (Zhu M. et al., 2009); (32) *Z Score*: a connectivity index based on degree distribution of a network (Rubinov and Sporns, 2010).

### Assessing the biological system profile for each studied target

The biological system profile for each studied target included: (1) the number of target-affiliated and target immediate-downstream signaling pathways in KEGG database (Kanehisa et al., 2017). The target-affiliated pathways were determined by considering that (a) the pathways of the studied target should be life-essential in both patients and healthy people and (b) the studied target should be in the pathway upstream with the capacity of regulating the biological function of the pathways. (2) The number of human tissues each target distributed in, assessed by the TissueDistributionDBs (Kogenaru et al., 2010) and Uniprot (UniProt Consortium, 2018) databases. A target was assumed to distribute in a given tissue if >5% of the total proteins are distributed in that tissue or the target concentration is higher than the average concentration of proteins in that tissue. (3) The number of human similarity proteins of a target outside the corresponding target family for probing off-target collateral effect (Zheng et al., 2006; Zhu F. et al., 2009). This was determined by BLAST similarity screening of human proteome in Uniprot database (UniProt Consortium, 2018) with a cutoff (*E-value* < 0.005; Song et al., 2006; Singh et al., 2007). (4) The differential expressions of the studied target in the disease-specific tissue between patients and healthy individuals (Li et al., 2018). The relevant data were collected directly from TTD (Li et al., 2018) and calculated based on the human gene expression raw data of *Affymetrix U133 Plus 2.0* platform in GEO (Barrett et al., 2013).

### Selecting the differential features indicating NTI drugs by artificial intelligence

The artificial intelligence (AI) has been recently proposed as a powerful technique for drug target discovery (Xu and Wang, 2014; Zhu et al., 2018), protein function prediction (Li et al., 2016a; Seo et al., 2018; Yu et al., 2018) and biomarker identification (Li B. et al., 2016; Li et al., 2017) through mimicking the human thinking procedures, learning processes and information extractions, which included the machine learning algorithm (Zhu et al., 2008a; Wang P. et al., 2015), the deep learning method (van der Burgh et al., 2017; Seo et al., 2018), and the cognitive-computing (Krittanawong et al., 2017). As one of the most popular machine learning algorithms, the *Boruta* algorithm based on wrapper method built around a random forest classifier (Kursa, 2014) was selected and adopted in this study. It is an extension to determine the relevance via comparing the relevance of the real features to that of the random probes (Pan et al., 2018). Since *Boruta* was constructed by an AI-based technique (machine learning), it was considered to be the most powerful approach with the stability in the variable selection, especially suitable for the low-dimensional dataset among other available strategies (Degenhardt et al., 2017). In this study, the differential features between NTI and NNTI drugs were therefore identified by *R package Boruta* (Shang et al., 2017). Particularly, human PPI network properties and biological system features of each target were first calculated, and the results of feature selection were then acquired using *R package Boruta* by setting the *p*-value < 0.05, maxRuns = 100, and doTrace = 2. In the meantime, the getImp was set to “getImpRfZ,” and the mcAdj and holdHistory were set to “TRUE.”

## Results and discussion

### Network properties and biological system profile of NTI and NNTI drugs

As reported, the human PPI network properties and biological system profile were key factors determining efficacy-safety balance (Zheng et al., 2006; Ragusa et al., 2010; Guo et al., 2018). Network properties were inherent feature of a target in the human PPI network, while biological system profile could reflect both the on-target and off-target pharmacology (Bender et al., 2007; Han et al., 2018; Zhu et al., 2018). Herein, 32 features of human PPI network together with 4 biological system properties were therefore adopted and calculated for further analyses. To the best of our knowledge, these were the most comprehensive sets of features ever applied for TI-related analysis. Table 1 listed the calculated values of ten properties based on the connectivity and adjacency in human PPI network. These connectivity/adjacency-based network properties were designed to describe the level of connectivity among human proteins or the neighborhood features of the studied proteins (Chen et al., 2016). The properties included bridging coefficient, clustering coefficient, degree, degree centrality, interconnectivity, neighbor connectivity, number of triangles, scaled degree, topological coefficient, and Z-score (corresponding definitions were provided in section Materials and Methods). As shown in Table 1, 8 (80.0%) out of 10 properties were significantly different (*p*-value < 0.05, highlighted by bold font) between the targets of NTI and NNTI drugs, and half of those 10 properties were with the most significant differences (*p*-value < 0.01, highlighted by bold-underline).

**Table 1:** The calculated values of 10 properties based on the connectivity and adjacency in the human PPI network.

**Connectivity/Adjacency based properties**	**Targets of the NTI drugs**		**Targets of the NNTI drugs**		***p*-values**
	**Mean ±SD**	**Median**	**Mean ±SD**	**Median**	
Bridging coefficient	5.62E−01 ± 1.44E+00	7.10E-02	3.72E+00 ± 9.02E+00	7.47E-01	2.15E-01
Clustering coefficient	1.07E−01 ± 1.67E−01	1.82E-02	4.06E−01 ± 4.06E−01	3.33E-01	**1.40E-02**
Degree	1.04E+01 ± 4.14*E*+00	1.10E+01	4.53E+00 ± 3.89E+00	3.00E+00	**3.56E-05**
Degree centrality	1.09E−03 ± 5.70E−04	1.00E-03	5.71E−04 ± 7.56E−04	0.00E+00	**2.90E-02**
Interconnectivity	2.59E−01 ± 1.04E−01	1.86E-01	5.89E−01 ± 1.44E−01	6.18E-01	**3.21E-07**
Neighbor connectivity	3.33E+01 ± 2.50E+01	2.79E+01	1.25E+01 ± 8.45E+00	1.13E+01	**1.57E-05**
Number of triangles	5.28E+00 ± 8.06E+00	1.00E+00	5.29E+00 ± 7.47E+00	3.00E+00	9.98E-01
Scaled degree	1.41E−02 ± 5.44E−03	1.50E-02	6.36E−03 ± 5.17E−03	4.00E-03	**7.01E-05**
Topological coefficient	1.67E−01 ± 1.53E−01	1.07E-01	3.41E−01 ± 2.42E−01	3.60E-01	**1.76E-02**
Z score	1.23E−03 ± 1.27E−02	3.00E-03	−1.63E−02 ± 1.22E−02	−2.20E-02	**1.17E-04**

*The mean values (together with standard deviation) and median values of these properties between the targets of NTI and NNTI drugs were provided, and the statistical difference (p-value) for each property between targets of NTI and NNTI drugs were also calculated (p-values <0.05 and <0.01 were highlighted by bold and bold-underline, respectively)*.

Similar to the connectivity/adjacency-based network property, the calculated values of 16 properties based on the shortest path length in the human PPI network were provided in Table 2 (corresponding definitions of these properties were provided in section Materials and Methods). As shown in Table 2, all properties were found to be significantly different (*p*-values < 0.05, in bold font) between the targets of NTI and NNTI drug, and 14 (87.5%) of the 16 properties were with the most significant difference (*p*-value < 0.01, bold-underline). Moreover, the calculated values of 4 human biological system properties were shown in Table 3 (definition of these properties was given in section Materials and Methods). As reported, these properties were frequently adopted to analyze the druggability of therapeutic targets for not only approved drugs but also the drugs in clinical trial development or withdrawn from market (Li et al., 2018). Herein, two properties were identified as significantly different (*p*-value < 0.01, bold-underline) between targets of NTI and NNTI drugs, which included the number of pathways affiliated by the targets of the studied drugs and the number of similarity proteins outside target's functional family. One thing needed to be emphasized was that the standard deviation of many properties was even larger than their mean value (such as bridging coefficient, clustering coefficient, and *Z*-score). These deviations indicated that the corresponding *p*-value may not be enough to measure the difference between the targets of NTI and NNTI drug. Moreover, any of the individual feature (*p*-value < 0.05 shown in Tables 1–3) could not be used to satisfactorily differentiate the targets of NTI drugs from that of the NNTI ones. Thus, this finding inspired us to discover the differential features using more advanced computational algorithm and collectively considering multiple properties.

**Table 2:** The calculated values of 16 properties based on the shortest path length in human PPI network.

**Shortest path length-based properties**	**Targets of the NTI drugs**		**Targets of the NNTI drugs**		***p*-values**
	**Mean ±SD**	**Median**	**Mean ±SD**	**Median**	
Average shortest path length	4.06E+00 ± 2.90E−01	3.95E+00	4.88E+00 ± 1.08E+00	5.09E+00	**1.06E-02**
Betweenness centrality	1.26E−03 ± 6.77E−04	1.77E-03	2.54E−04 ± 3.94E−04	1.09E-05	**1.59E-08**
Average closeness centrality	2.47E−01 ± 1.63E−02	2.53E-01	1.97E−01 ± 2.26E−02	1.92E-01	**5.31E-07**
Current flow betweenness	3.07E−03 ± 1.35E−03	4.00E-03	8.57E−04 ± 1.17E−03	5.00E-04	**3.38E-06**
Deviation	1.11E+04 ± 2.31E+03	1.03E+04	1.96E+04 ± 4.39E+03	2.03E+04	**4.24E-06**
Distance deviation	6.17E+03 ± 2.02E+03	6.93E+03	4.30E+03 ± 2.37E+03	4.16E+03	**1.57E-02**
Distance sum	3.23E+04 ± 2.31E+03	3.14E+04	4.08E+04 ± 4.39E+03	4.15E+04	**4.24E-06**
Eccentric	1.11E+00 ± 4.27E−01	1.34E+00	5.97E−01 ± 4.14E−01	3.40E-01	**6.09E-04**
Eccentricity	1.02E+01 ± 4.27E−01	1.00E+01	1.14E+01 ± 7.45E−01	1.10E+01	**6.44E-05**
Eccentricity centrality	9.79E−02 ± 3.85E−03	1.00E-01	8.84E−02 ± 5.79E−03	9.10E-02	**2.30E-05**
Harmonic closeness centrality	2.10E+03 ± 1.53E+02	2.14E+03	1.64E+03 ± 2.03E+04	1.59E+03	**3.95E-07**
Load centrality	1.35E−03 ± 7.83E−04	2.00E-03	2.86E−04 ± 4.69E−04	0.00E+00	**3.75E-07**
Normalized betweenness	2.81E−03 ± 1.53E−03	4.00E-03	5.71E−04 ± 8.52E−04	0.00E+00	**2.53E-08**
Residual closeness centrality	6.00E+02 ± 1.05E+02	6.29E+02	3.07E+02 ± 1.23E+02	2.74E+02	**1.32E-07**
Radiality	8.09E−01 ± 1.81E−02	8.16E-01	7.43E−01 ± 3.33E−02	7.44E-01	**1.21E-06**
Stress	1.56E+06 ± 9.11E+05	2.24E+06	3.04E+05 ± 4.82E+05	4.69E+03	**2.13E-08**

*Mean values (together with standard deviation) and median values of these properties between the targets of NTI and NNTI drugs were provided, and the statistical difference (p-value) for each property between targets of NTI and NNTI drugs were also calculated (p-values <0.05 and <0.01 were highlighted by bold and bold-underline, respectively)*.

**Table 3:** The calculated values of four human biological system properties.

**Human biological system properties**	**Targets of the NTI drugs**		**Targets of the NNTI drugs**		***p*-values**
	**Mean ±SD**	**Median**	**Mean ±SD**	**Median**	
No. of pathways affiliated by the primary therapeutic target	6.10 ± 1.80	7.00	1.14 ± 0.38	1.00	**2.50E-15**
No. of similarity proteins outside the target family	24.4 ± 15.22	29.00	11.79 ± 6.21	11.00	**1.46E-05**
Differential expression levels between patients and healthy individuals	0.42 ± 0.35	0.56	0.33 ± 0.32	0.20	3.86E-01
No. of tissues distributed by the primary therapeutic target	3.38 ± 0.81	3.00	3.61 ± 1.82	3.00	6.06E-01

*The mean values (together with standard deviation) and median values of these properties between the targets of NTI and NNTI drugs were provided, and the statistical difference (p-value) for each property between targets of NTI and NNTI drugs were also calculated (p-values <0.05 and <0.01 were highlighted by bold and bold-underline, respectively)*.

### Discovering the key features of NTI drug targets by artificial intelligence

Based on the in-depth investigation of 36 properties in Tables 1–3, several properties were found to be not fully independent or even duplicate in their descriptions (like degree vs. scaled degree). In this study, all 36 properties were systematically reviewed, and 19 of these 36 were identified to be substantially overlapped with some other properties (Table 4). Since there was significant dependence among the 19 properties, the use of all 36 properties for statistical feature selection may introduce strong biases. Thus, the 19 properties were grouped based on their innate mutual dependence. As shown in Table 4, five property groups were generated by considering equation and description of these 19 properties, and each group was named by the first property (ordered alphabetically) in the corresponding group. As a result, these five groups included: the *average closeness centrality, average shortest path length, betweenness centrality, degree, eccentricity*. To minimize the possible bias induced by the innate mutual dependence among properties, only these five properties were considered in subsequent feature selection analysis, instead of investigating all 19 properties. Taking the remaining 17 relatively independent properties into consideration, 22 properties in total of each target were selected for subsequent feature selection.

**Table 4:** 19 substantially overlapped network properties grouped into 5 property groups based on their innate mutual dependence.

**Property group**	**Original property**	**Equation of the property**	**Description of the property**
Average closeness centrality	Average closeness centrality	$1/\left(\frac{1}{N}\sum_{j=1}^{N}D_{ij}\right)$	The average number of steps required to reach the studied node from any node in the network
	Harmonic closeness centrality	$\sum_{j=1}^{N}\frac{1}{D_{ij}}$	The sum of the reciprocals of the average shortest path lengths of each node in the network
	Residual closeness centrality	$\sum_{j=1}^{N}\frac{1}{2^{D_{ij}}}$	The closeness measured by removing the studied node
	Sum closeness centrality	$1/\sum_{j=1}^{N}D_{ij}$	The reciprocal of the sum of the shortest paths between the studied node and all other nodes in the network
Average shortest path length	Average shortest path length	$\frac{1}{N-1}\sum_{j=1}^{N}D_{ij}$	The average length of the shortest paths between the studied node and all other nodes in network
	Deviation	*distSum*_*i*_−*unipolarity*_*i*_	The variation between the total sum of node distances and the network unipolarity
	Distance sum	$\sum_{j=1}^{N}D_{ij}$	The sum of all shortest paths starting from the studied node
Betweenness centrality	Betweenness centrality	$\sum_{s\neq i\neq t}\frac{\sigma_{st}\left(i\right)}{\sigma_{st}}$	The number of times the studied node serving as a linking bridge along the shortest paths between any two nodes
	Current flow betweenness	$\frac{1}{N_{b}}\sum_{s,t\in V}\tau_{st}\left(i\right)$	A centrality index measuring the level of information travels along all possible paths within the network
	Current flow closeness	$\frac{N_{C}}{\sum_{s\neq t}p_{st}\left(s\right)-p_{st}\left(t\right)}$	The variant of current flow betweenness
	Load centrality	∑_*s*≠*i*≠*t*_σ_*st*_(*i*)	The fraction of all the shortest paths that pass through the studied node
	Normalized betweenness centrality	$\frac{cenBtw_{i}-min\left(cenBtw_{G}\right)}{max\left(cenBtw_{G}\right)-min\left(cenBtw_{G}\right)}$	The fraction of network shortest paths that the studied protein lies on
Degree	Degree	*Degree*_*i*_	The total number of edges linked to a node
	Degree centrality	$\frac{deg_{i}}{N-1}$	The number of links incident upon the studied node
	Number of self-loops	*Selfloop*_*i*_	The number of edges starting and ending at the same node
	Scaled degree	$\frac{deg_{i}}{max\left(deg_{G}\right)}$	The degree of the studied node relative to the most connected node within the same module
	Z score	[*deg*_*i*_−*avg*(*deg*_*G*_)]/*dev*(*deg*_*G*_)	A connectivity index based on the degree distribution of network
Eccentricity	Eccentricity	*max*(*D*_*ij*_)	The maximum non-infinite shortest path length between the studied node and all other nodes in the network
	Eccentricity centrality	1/*max*(*D*_*ij*_)	The largest geodesic distance between the node and any other nodes

**Figure 1:**
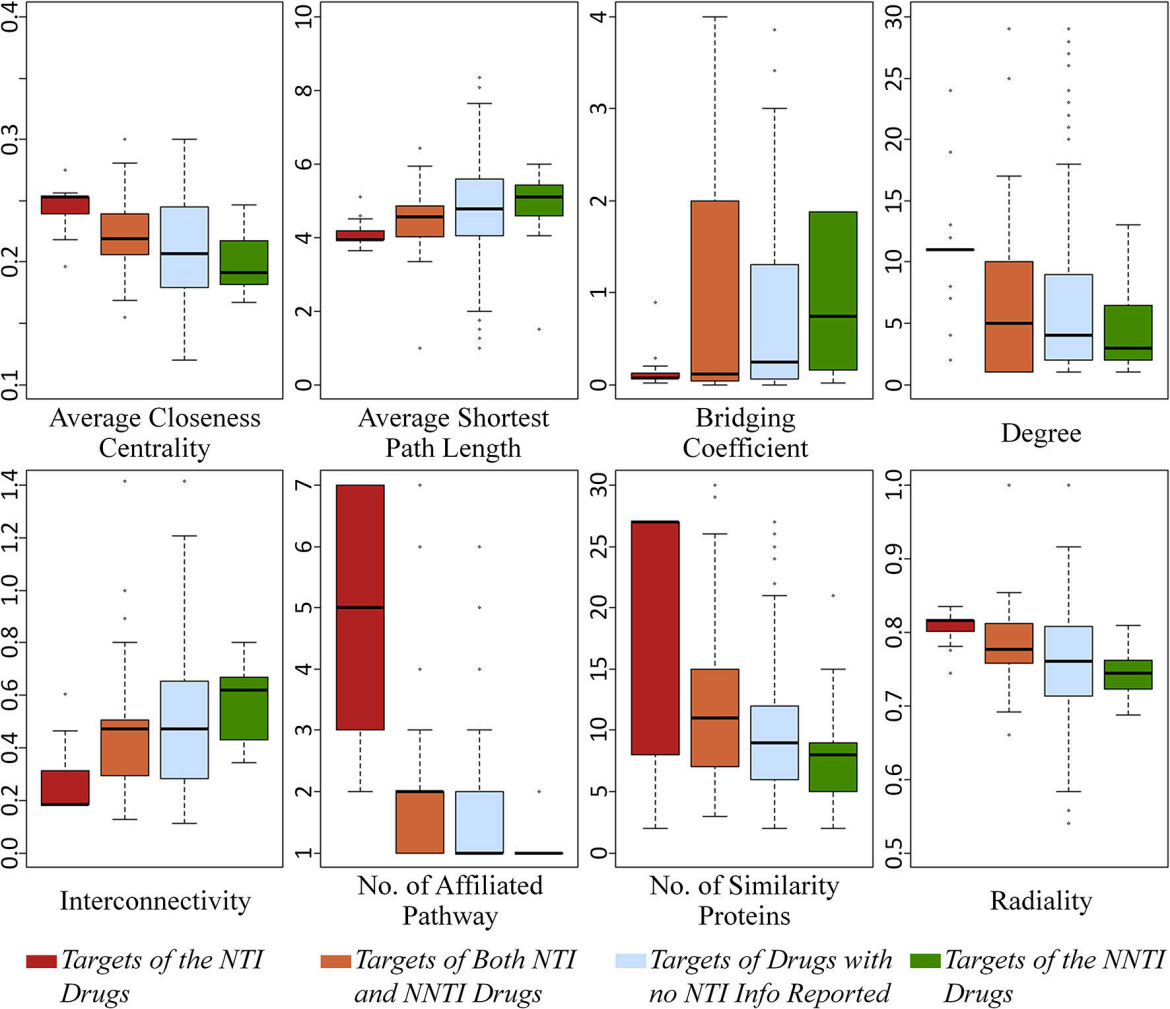
Boxplots of eight key features identified in this study. For each feature, there were four plots colored in red, orange, light blue and green which indicated the targets of NTI drugs, both NTI and NNTI drugs, drugs with no NTI data reported and NNTI drugs, respectively.

As one of the most popular feature selection strategies based on AI, the *Boruta* algorithm based on a wrapper method built around a random forest classifier (Kursa, 2014) was adopted in this study. *Boruta* was considered the most powerful method with the stability in variable selection, especially suitable for the low-dimensional dataset among other reported strategies (Degenhardt et al., 2017). In this study, the key differential features were thus selected from 22 properties using *R package Boruta* by setting the *p*-value < 0.05. As a result, eight properties were selected as able to collectively reflect the target's mechanism underlying NTI drugs. As illustrated in Figure 1, the boxplots colored in red and green referred to the targets of NTI and NNTI drugs, respectively. Some key features increased from the targets of NTI drug to that of NNTI one (such as *average shortest path length*), while others demonstrated a decrease (such as *average closeness centrality*). Based on the comprehensive literature review, some of those 8 key features had been reported to be indirectly relevant to drugs' efficacy-safety balances. For example, the lower value of *average closeness centrality* of target was reported to demonstrate a less lethality risk (Chen et al., 2011), which was consistent with the findings of this study (a much higher *average closeness centrality* of the targets of NTI drugs was observed compared with that of NNTI ones, shown in Figure 1). Moreover, the higher level (lower value) of *interconnectivity* was frequently observed in lethal diseases such as cardiovascular disorder and cancer (Muhammd et al., 2018). Oncological and cardiovascular disorder had been recognized as life-threatening diseases, and the majority of their drugs were reported to be NTI ones (Muller and Milton, 2012; Yu et al., 2015). Thus, the result of *interconnectivity* in Figure 1 was consistent with these previous reports, which further validated the effectiveness of applied algorithm in identifying key target features underlying NTI drugs.

Moreover, there were four groups of targets as defined in section Materials and Methods: (a) targets of NTI drugs, (b) targets of both NTI and NNTI drugs, (c) targets of drugs without reported TI, and (d) targets of NNTI drugs. Apart from the target groups (a) and (d), the remaining groups provided more complicated and informative data for illustrating the mechanism underlying NTI drugs. On one hand, the targets in group (b) were affected by both NTI and NNTI drugs, which might reflect properties from both sides, but might also be significantly affected by the properties of confirmed NTI drugs. On the other hand, no TI data of the group (c) targets was reported based on literature review. It was possible that some NTI drugs were not discovered for those targets. But considering the large number of group (c) targets (339 in total), it was highly possible that most of those group (c) targets were only aimed by NNTI drugs, and just a small fraction of which could find new NTI drug in the future. The value of 8 properties of those 4 target groups were illustrated in Figure 1. It was interesting that all properties followed a clear descending/ascending trend from the targets of group (a) to (d), which was in accordance with the analyses provided above. Thus, these findings could be another line of evidence that validated the effectiveness of the feature identification algorithm applied in this study.

### Target mechanism underlying NTI drugs collectively determined by multiple profiles

By collectively considering Figure 1 and Tables 1–3, seven out of those eight selected key features showed significant difference (*p*-value < 0.05), but it was clear that these significant differences did not guarantee the corresponding feature as the key differential one (57.7% of the features with significant difference (*p*-value < 0.05) were not selected as key differential ones). Moreover, significant difference was not observed for the selected key feature *bridging coefficient* (*p*-value = 0.22). This finding indicated that those eight features collectively determined the target mechanism of NTI drugs, and the TI-related mechanism might be the result of the synergistical effects among those features. Moreover, the majority of these eight key features were identified for the first time by this study, and this work was also the first analysis on the collective effects of both PPI network properties and biological system profile on the drug efficacy-safety balance.

**Figure 2:**
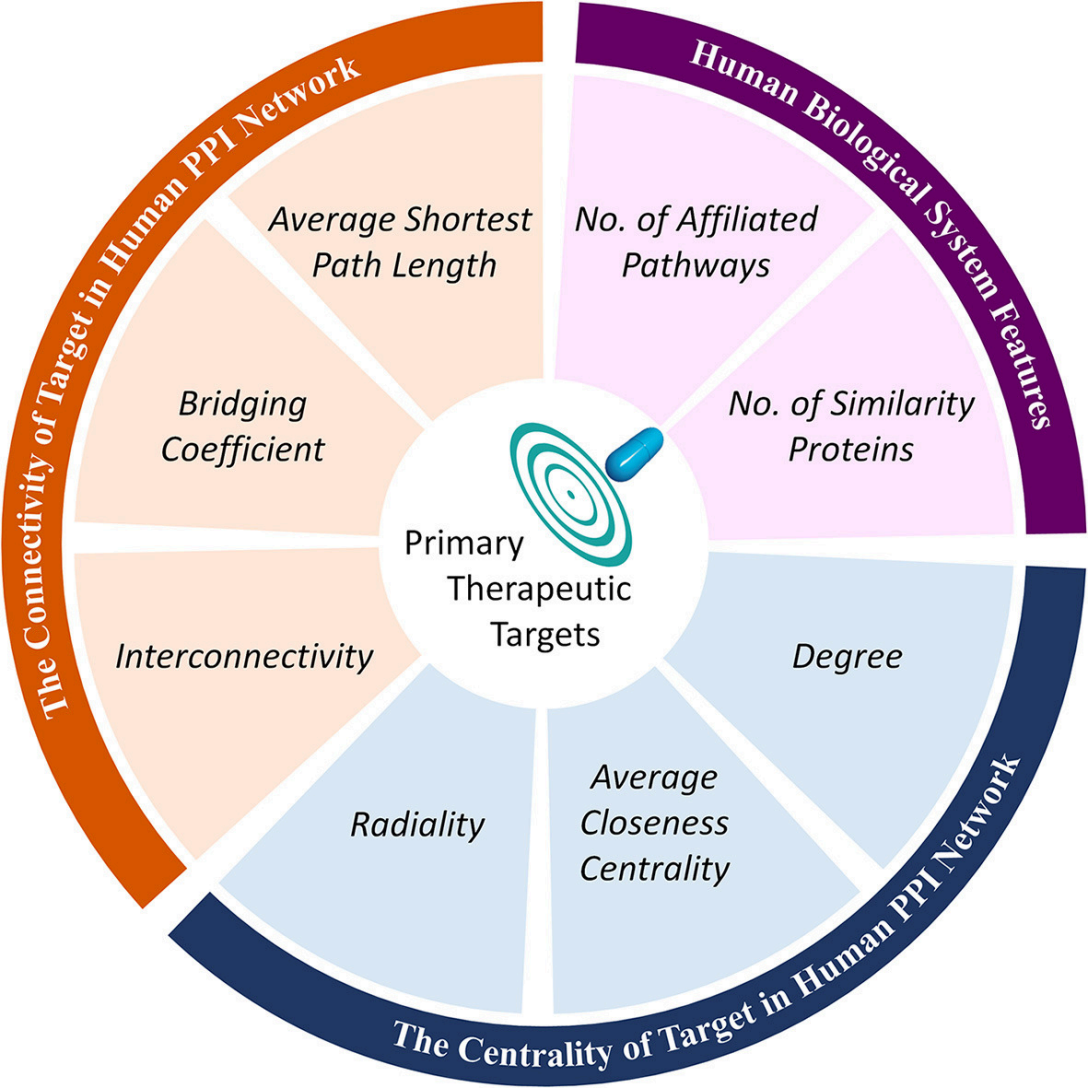
Classification of eight key features identified in this study into three feature groups.

Further analysis on these eight identified key features (shown in Figure 1) revealed that these key features were found to belong to three feature groups. These feature groups were connectivity and centrality of targets in human PPI network together with human biological system features. By combining the data in Figure 1, the key features within the same feature group (illustrated in Figure 2) followed the same ascending/descending trends, which were colored by the same background. As shown in Figure 2, the targets of NTI drugs were highly centralized and connected, and the number of similarity proteins and the number of affiliated pathways were substantially higher than those of NNTI drug. Since the number of similarity proteins and affiliated pathways was reported to be good indicator of target druggability (Zhu F. et al., 2009; Li et al., 2018), the NTI profile identified in this study was in accordance with that of reported target druggability.

## Conclusion

This work is the first study conducting comprehensive review on the TI data of all FDA approved drugs (Supplementary Table S1) and revealing the collective effects of both human PPI network properties and biological system profiles on drug efficacy-safety balance. Eight key features were identified here as collectively differentiating the target mechanisms between NTI and NNTI drugs. These features revealed that the targets of NTI drugs were highly centralized and connected in human PPI network, and the numbers of similarity proteins and target-affiliated pathways were both much higher than those of NNTI drugs. These findings together with the newly discovered features/feature groups clarified the key factors indicating drug's narrow TI and could therefore provide a novel direction for determining the delicate drug efficacy-safety balance.

## Author contributions

FZ conceived the idea and supervised the work. XL, JY, and JT performed the research. XL, JY, JT, YL, QY, ZX, RZ, YW, JH, LT, and WX prepared and analyzed the data. FZ wrote the manuscript. All authors have read and approved this manuscript.

### Conflict of interest statement

The authors declare that the research was conducted in the absence of any commercial or financial relationships that could be construed as a potential conflict of interest.
