# Supplementary material for: Determining the Balance Between Drug Efficacy and Safety by the Network and Biological System Profile of Its Therapeutic Target
Source: Front Pharmacol. 2018 Oct 31;9:1245. doi: 10.3389/fphar.2018.01245 (PMC6220079; doi:10.3389/fphar.2018.01245)
Supplement: Supplementary file 1 [file Table_1.pdf]

Supplementary Information for:

Determining the Balance between Drug Efficacy and Safety by the Network and Biological System Profile of Its Therapeutic Target

Xiaoxu LI[1,2], Jiayi YIN[1], Jing TANG[1,2], Yinghong LI[1,2], Qingxia YANG[1,2], Ziyu XIAO[1], Runyuan ZHANG[1], Yunxia WANG[1], Jiajun HONG[1], Lin TAO[3], Weiwei XUE[2], Feng ZHU[1,2,*]

[1] College of Pharmaceutical Sciences, Zhejiang University, Hangzhou 310058, China

[2] School of Pharmaceutical Sciences and Collaborative Innovation Center for Brain Science, Chongqing University, Chongqing 401331, China

[3] Key Laboratory of Elemene Class Anti-cancer Chinese Medicine of Zhejiang Province, School of Medicine, Hangzhou Normal University, Hangzhou, P. R. China 310036

*Correspondence should be sent to Prof. Feng Zhu (zhufeng@zju.edu.cn; prof.zhufeng@gmail.com)

Table S1. 161 NTI (**Y**) and 29 NNTI (**N**) drugs approved by FDA together with their disease indications and corresponding targets.

FDA Approved Drug	Time of Approval	FDA Approved Representative Indication(s)	Target Name	TI Information and Reference(s)
Apixaban	2012	Stroke and systemic embolism; Deep vein thrombosis	Coagulation factor X	**N**[1,2]
Aripiprazole	2002	Schizophrenia	Dopamine D2 receptor	**N**[3]
Atomoxetine HCl	2002	Attention-deficit hyperactivity disorder	Norepinephrine transporter	**N**[4]
Clobazam	2011	Seizures associated with lennox–gastaut syndrome	GABA(A) receptor	**N**[1,5]
Clonazepam	1975	Seizure disorders; Panic disorder	GABA(A) receptor	**N**[1]
Enalapril Maleate	1985	Hypertension	Angiotensin-converting enzyme	**N**[6]
Ethosuximide	1960	Epilepsy	VG calcium channel alpha-1G	**N**[1]
Ezogabine	2011	Partial-onset seizures	KQT-like 2/3	**N**[1,5]
Felbamate	1993	Epilepsy	GABA(A)/NMDA receptor	**N**[1]
Gabapentin	1993	Postherpetic neuralgia; Partial onset seizures	VG calcium channel A2D2/A2D3	**N**[7]
Lacosamide	2008	Partial-onset seizures in epilepsy	Dystrophin-related protein 2	**N**[1,8]
Lamivudine	1995	Acquired immune deficiency syndrome	HIV reverse transcriptase	**N**[9]
Lamotrigine	1994	Bipolar disorder; Epilepsy	Voltage-gated sodium channel	**N**[1]
Levetiracetam	1999	Partial onset seizures in adults with epilepsy	Synaptic vesicle glycoprotein 2A	**N**[1]
Linagliptin	2011	Type 2 diabetes	Dipeptidyl peptidase IV	**N**[10]
Mechlorethamine HCl	1949	T-cell lymphoma	Human DNA	**N**[11]
Mitoxantrone HCl	1987	Multiple sclerosis	DNA topoisomerase II	**N**[12]
Montelukast Sodium	1998	Asthma; Seasonal allergic rhinitis	Leukotriene CysLT1 receptor	**N**[13]

Oxcarbazepine	2000	Epileptic seizures	Voltage-gated sodium channel	**N**[1]
Perampanel	2012	Partial-onset seizures in patients with epilepsy	AMPA receptor	**N**[1,2]
Pimecrolimus	2001	Mild to moderate atopic dermatitis	Calcineurin	**N**[14]
Pregabalin	2004	Neuralgia; Partial onset seizures; Fibromyalgia	VG calcium channel alpha-2	**N**[1,15]
Rivaroxaban	2011	Stroke and systemic embolism; Deep vein thrombosis	Coagulation factor X	**N**[1,5]
Rolapitant HCl	2015	Nausea and vomiting associated with chemotherapy	Neurokinin-1 receptor	**N**[16]
Rufinamide	2008	Seizures associated with lennox–Gastaut syndrome	Target Not Clear	**N**[1,8]
Topiramate	1996	Partial onset seizures	GABA(A) receptor	**N**[1]
Vigabatrin	2009	InfAntile spasms and complex partial seizures in adults	GABA transaminase	**N**[1,17]
Zonisamide	2000	Partial seizures in adults with epilepsy	VG sodium channel 1 subunit alpha	**N**[1]
Amikacin	1981	Bacterial infections	Bacterial 30S ribosome	**Y**[18]
Amiodarone HCl	1985	Recurrent ventricular arrhythmias	Target Not Clear	**Y**[19]
Amitriptyline HCl	1961	Major depressive disorder	Serotonin transporter	**Y**[20]
Amlodipine Besylate	1992	Hypertension; Coronary artery disease	Target Not Clear	**Y**[21]
Amphotericin B	1964	Fungal infections	Fungal ergosterol	**Y**[22]
Argatroban	2000	Thrombosis	Coagulation factor II	**Y**[1]
Axitinib	2012	Advanced renal cell carcinoma	VEGF-2 receptor	**Y**[23]
Azathioprine	1968	Rheumatoid arthritis	Amidophosphoribosyl transferase	**Y**[24]
Baclofen	1977	Multiple sclerosis	GABA(B) receptor	**Y**[25]
Busulfan	1954	Chronic myelogenous leukemia	Human DNA	**Y**[26]

Capecitabine	1998	Breast cancer	Thymidylate synthase	**Y**[27]
Carbamazepine	1968	Epilepsy; Trigeminal neuralgia	Sodium channel SCN5A	**Y**[1,28,29]
Carboplatin	1989	Ovarian cancer	Human DNA	**Y**[30]
Chloroquine	1949	Malaria; Rheumatoid Arthritis	Plasmodium lactate dehydrogenase	**Y**[31]
Cidofovir	1996	Cytomegalovirus	Viral DNA polymerase	**Y**[32]
Cisapride	1993	Gastroesophageal reflux disease	5-HT 4 receptor	**Y**[33]
Cisplatin	1978	Metastatic testicular, bladder and ovarian tumors	Human DNA	**Y**[34]
Clindamycin	1970	Susceptible anaerobic bacteria infections	Bacterial 50S ribosome subunit	**Y**[1,35]
Clonidine	1974	Hypertension	Alpha-2 adrenergic receptor	**Y**[1,35,36]
Clozapine	1989	Resistant schizophrenia	5-HT 2a receptor	**Y**[37]
Codeine	2009	Mild to moderately severe pain	Opioid receptors	**Y**[38]
Colchicine	2009	Gout flares; Familial mediterranean fever	Tubulin	**Y**[39,40]
Colistin	1962	Gram-negative bacilli infections	Bacterial cell membrane	**Y**[41]
Cyclophosphamide	1959	Malignant diseases; Minimal change nephrotic syndrome	Human DNA	**Y**[42]
Cyclosporine	1983	Chronic rejection	Calcineurin	**Y**[1,43]
Dalteparin Sodium	1994	Prophylaxis of deep vein thrombosis	Coagulation factor X	**Y**[44]
Digitoxin	1982	Heart failure	Na+/K+ ATPase	**Y**[1,45]
Digoxin	1954	Mild to moderate heart failure; Chronic atrial fibrillation	Na+/K+ ATPase	**Y**[1,46]
Disopyramide	1977	Ventricular arrhythmias	Sodium channel SCN5A	**Y**[47]
Docetaxel	1996	Breast cancer; NSCLC; HRPC	Tubulin beta-3 chain	**Y**[48]

Doxorubicin HCl	1974	Oncology	DNA topoisomerase II	**Y**[49]
Efavirenz	1998	HIV Infection	HIV reverse transcriptase	**Y**[50]
Epinephrine	1951	Anaphylaxis; Cardiac arrest	Alpha-2 adrenergic receptor	**Y**[51]
Epirubicin HCl	1999	Axillary node tumor	DNA topoisomerase II	**Y**[52]
Ethinyl Estradiol	1943	Contraception; Menopausal hormone therapy	Estrogen receptor	**Y**[1]
Etoposide	1983	Refractory testicular tumors; Small cell lung cancer	DNA topoisomerase II	**Y**[53]
Everolimus	2009	Renal cell carcinoma	mTOR	**Y**[1,54]
Fentanyl	1968	Analgesia	Opioid receptor mu	**Y**[38]
Flecainide Acetate	1985	Arrhythmic	Sodium channel SCN5A	**Y**[55]
Fluorouracil	1962	Colon and rectum, breast, Gastric, Pancreatic tumor	Thymidylate synthase	**Y**[56]
Fondaparinux Sodium	2001	Deep vein thrombosis prophylaxis	Coagulation factor X	**Y**[57]
Fosphenytoin Sodium	1996	Epilepsy; convulsions	Sodium channel SCN5A	**Y**[58]
Gefitinib	2003	Non-small-cell lung cancer	Epidermal growth factor receptor	**Y**[59]
Gemcitabine HCl	1996	Pancreatic Cancer	Ribonucleotide reductase	**Y**[60]
Gentamicin	1970	Antibacterial	Bacterial 30S ribosome subunit	**Y**[18]
Guanethidine	1960	Hypertensive emergency.	Norepinephrine transporter	**Y**[1,35]
Heparin Sodium	1939	Anticoagulant	Antithrombin III	**Y**[61]
Hydrocodone	2013	Moderate to severe pain; Cough	Opioid receptor mu	**Y**[38]
Hydroxyurea	1967	Sickle-cell anemia	Ribonucleotide reductase	**Y**[62]
Imipramine HCl	1959	Major depressive disorder; Childhood enuresis	Target Not Clear	**Y**[63]

Indinavir Sulfate	1996	HIV Infection	HIV protease	**Y**[20]
Insulin	2000	Diabetes mellitus	Insulin	**Y**[64]
Interferon Alfa-2b	1986	Malignant melanoma	Interferon-alpha/beta receptor 2	**Y**[65]
Interferon Alfa-n3	1989	Genital warts	Interferon-alpha/beta receptor 2	**Y**[65]
Irinotecan HCl	1996	Metastatic carcinoma of the colon or rectum	DNA topoisomerase I	**Y**[49]
Ivermectin	1996	Nondisseminated intestinal threadworm	Parasitic Glu-chloride channel	**Y**[66]
Kanamycin	1973	Antibacterial	Bacterial 30S ribosome subunit	**Y**[67]
Levamisole HCl	1990	Parasitic worm infections	Parasitic worm nicotinic AChR	**Y**[68]
Levothyroxine	2000	Thyroid disease, Hypothyroidism	Thyroid hormone receptor beta	**Y**[1,46]
Lidocaine	1948	Local anesthetic; Ventricular arrhythmias; Epilepsy	Voltage-gated sodium channel	**Y**[69]
Lindane	1974	Infestations of lice and scabies	GABA(A) receptor	**Y**[70]
Linezolid	2000	Pneumonia; Skin and skin structure infections	Bacterial 50S ribosome subunit	**Y**[71]
Lithium Carbonate	1970	Bipolar disorder	Target Not Clear	**Y**[72]
Lithium Citrate	1980	Manic episodes; bipolar disorder	Glycogen synthase kinase-3	**Y**[73]
Loperamide HCl	1976	Diarrhea	Opioid receptor mu	**Y**[66]
Mercaptopurine	1953	Lymphatic leukemia; Acute myelogenous leukemia	IMP dehydrogenase	**Y**[74]
Methadone	1947	Dry cough; Opioid type drug dependence	Opioid receptor mu	**Y**[38]
Methotrexate	1953	Neoplastic diseases; Psoriasis; Rheumatoid arthritis	Dihydrofolate reductase	**Y**[30]
Midazolam HCl	1985	Anesthesia	Translocator protein	**Y**[75]
Mirtazapine	1996	Depression	5-HT 2c receptor	**Y**[76]

Mitomycin	1981	anticancer	Human DNA	**Y**[77]
Mitotane	1970	Inoperable, adrenal cortical carcinoma	Estrogen receptor	**Y**[78]
Morphine	1984	Severe pain	Opioid receptor mu	**Y**[38]
Mycophenolic Acid	1995	Organ rejection inpatients after heart transplants	IMP dehydrogenase	**Y**[79]
Neomycin	1964	Bacterial infections	Bacterial 30S ribosome subunit	**Y**[18]
Nortriptyline HCl	1964	Major depressive disorder	Target Not Clear	**Y**[4]
Olanzapine	1996	Psychotic disorders	Dopamine D2 receptor	**Y**[80]
Orphenadrine HCl	1957	Parkinson's disease	NMDA receptor	**Y**[81]
Oxaliplatin	2002	Colon or rectum carcinomas	Human DNA	**Y**[82]
Oxymorphone	1959	Severe pain; Preoperative medication	Opioid receptor mu	**Y**[38]
Oxytocin	1980	Induction of labor	Oxytocin receptor	**Y**[83]
Paclitaxel	1992	Kaposi's sarcoma	Tubulin beta	**Y**[84]
Pazopanib HCl	2009	Renal cell carcinoma	VEGF-2 receptor	**Y**[23]
Pemetrexed	2004	Malignant pleural mesothelioma	Dihydrofolate reductase	**Y**[85]
Pentobarbital	1973	Insomnia	GABA(A) receptor	**Y**[86]
Phenprocoumon	1957	Thrombosis	Vitamin K epoxide reductase	**Y**[87]
Phenytoin	1953	Seizures	Voltage-gated sodium channel	**Y**[1,58]
Pimozide	1984	Tourette’s Disorder	Dopamine D2 receptor	**Y**[88]
Polymyxin B	1964	Bacterial infections	Target Not Clear	**Y**[41]
Prazosin HCl	1976	Hypertension	Alpha 1A adrenoceptor	**Y**[1,35]

Primidone	1954	Epilepsy	Voltage-gated sodium channel	**Y**[1,35]
Procainamide HCl	1950	Ventricular arrhythmias	BTX-activated cardiac channels	**Y**[89]
Propafenone HCl	1989	Episodic atrial fibrillation; Ventricular arrhythmias	Beta adrenoceptor	**Y**[1]
Propofol	1989	Sedation; Anesthesia	GABA(A) receptor	**Y**[90]
Pyrazinamide	1971	Tuberculosis	Mycobacterium tuberculosis InhA	**Y**[91]
Quinidine	1950	Ventricular arrhythmias	Sodium channel SCN5A	**Y**[1]
Quinine	2005	Uncomplicated plasmodium falciparum malaria	Target Not Clear	**Y**[92]
Regorafenib	2012	Hepatocellular and Colorectal cancer	c-Kit; KDR	**Y**[23]
Ribavirin	1985	Hepatitis C	IMP dehydrogenase	**Y**[93]
Secobarbital	1950	Intractable insomnia	GABA(A) receptor	**Y**[86]
Sirolimus	1999	Organ rejection in patients receiving renal transplants	mTOR	**Y**[1,94]
Sorafenib Tosylate	2005	Hepatocellular carcinoma; Renal cell carcinoma	c-RAF; B-Raf; c-Kit; PDGFRB; KDR	**Y**[23]
Sotalol HCl	1992	Ventricular arrhythmias	Beta-adrenergic receptor	**Y**[1]
Streptomycin	1946	Bacterial infections	Bacterial 30S ribosome subunit	**Y**[18]
Sunitinib Malate	2006	Gastrointestinal stromal tumor; renal cell carcinoma	c-Kit; PDGFRB; KDR	**Y**[23]
Tacrolimus	1994	Prophylaxis of organ rejection	Calcineurin	**Y**[1,95]
Teniposide	1992	Acute lymphocytic leukemia	DNA topoisomerase II	**Y**[30]
Theophylline	1970	Chronic obstructive pulmonary disease; Asthma	Phosphodiesterase 4	**Y**[1,96]
Thioguanine	1966	Acute nonlymphocytic leukemia	Human DNA	**Y**[74]
Thioridazine HCl	1962	Schizophrenic	Dopamine D2 receptor	**Y**[55]

Tizanidine HCl	1996	Muscle spasticity	Alpha-2 adrenergic receptor	**Y**[97]
Tobramycin Sulfate	1975	Bacterial infection	Bacterial 16S ribosome subunit	**Y**[98]
Tolbutamide	1961	Diabetes mellitus	Sulfonylurea receptor 1	**Y**[99]
Topotecan HCl	1996	Ovarian cancer	DNA topoisomerase I	**Y**[30]
Tramadol HCl	1995	Pain	Opioid receptor mu	**Y**[38]
Valproic Acid	1978	Epilepsy	Histone deacetylase	**Y**[1,29]
Vancomycin	1964	Infections caused by staphylococci	Bacterial cell wall synthesis	**Y**[100]
Vandetanib	2011	Unresectable or metastatic medullary thyroid cancer	EGFR; KDR	**Y**[23]
Vinblastine Sulfate	1965	Lymphoma	Tubulin	**Y**[55]
Vincristine Sulfate	1963	Leukemia; Neuroblastoma; Small cell lung cancer	Tubulin	**Y**[101]
Vinorelbine Tartrate	1994	Non-small cell lung cancer	Tubulin beta-3 chain	**Y**[102]
Voriconazole	2002	Fungal infection	Fungal Cytochrome P450LI	**Y**[103]
Warfarin Sodium	1954	Venous thrombosis; Pulmonary embolism	Vitamin K epoxide reductase	**Y**[46]

References:

1 E. Dahmane; J. Gobburu; V. Ivaturi. Pharmacometric Approach To Define Narrow Therapeutic Index (NTI) Drugs & Evaluate Bioequivalence (BE) Criteria for NTI Drugs. Drugs@FDA. 2018, 1(1): 1-25

2 A. Mullard. 2012 FDA drug approvals. Nat Rev Drug Discov. 2013, 12(2): 87-90

3 S. D. Carstairs; S. R. Williams. Overdose of aripiprazole, a new type of antipsychotic. J Emerg Med. 2005, 28(3): 311-3

4 T. Spencer; J. Biederman; T. Wilens. Nonstimulant treatment of adult attention-deficit/hyperactivity disorder. Psychiatr Clin North Am. 2004, 27(2): 373-83

5 A. Mullard. 2011 FDA drug approvals. Nat Rev Drug Discov. 2012, 11(2): 91-4

6 R. K. Verbeeck; I. Kanfer; R. Lobenberg; B. Abrahamsson; R. Cristofoletti; D. W. Groot; P. Langguth; J. E. Polli; A. Parr; V. P. Shah; M. Mehta; J. B. Dressman. Biowaiver Monographs for Immediate-Release Solid Oral Dosage Forms: Enalapril. J Pharm Sci. 2017, 106(8): 1933-43

7 K. S. Walia; E. A. Khan; D. H. Ko; S. S. Raza; Y. N. Khan. Side effects of antiepileptics--a review. Pain Pract. 2004, 4(3): 194-203

8 B. Hughes. 2008 FDA drug approvals. Nat Rev Drug Discov. 2009, 8(2): 93-6

9 S. Strauch; E. Jantratid; J. B. Dressman; H. E. Junginger; S. Kopp; K. K. Midha; V. P. Shah; S. Stavchansky; D. M. Barends. Biowaiver monographs for immediate release solid oral dosage forms: lamivudine. J Pharm Sci. 2011, 100(6): 2054-63

10 U. Graefe-Mody; S. Retlich; C. Friedrich. Clinical pharmacokinetics and pharmacodynamics of linagliptin. Clin Pharmacokinet. 2012, 51(7): 411-27

11 J. W. Eklund; S. Trifilio; M. F. Mulcahy. Chemotherapy dosing in the setting of liver dysfunction. Oncology. 2005, 19(8): 1057-63; discussion 63-4, 69

12 S. Saletan. Mitoxantrone: an active, new antitumor agent with an improved therapeutic index. Cancer Treat Rev. 1987, 14(3-4): 297-303

13 B. Knorr; L. M. Franchi; H. Bisgaard; J. H. Vermeulen; P. LeSouef; N. Santanello; T. M. Michele; T. F. Reiss; H. H. Nguyen; D. L. Bratton. Montelukast, a leukotriene receptor antagonist, for the treatment of persistent asthma in children aged 2 to 5 years. Pediatrics. 2001, 108(3): E48

14 L. F. Eichenfield; L. Beck. Elidel (pimecrolimus) cream 1%: a nonsteroidal topical agent for the treatment of atopic dermatitis. J Allergy Clin Immunol. 2003, 111(5): 1153-68

15 N. A. Author. Hot drugs 2004. Nat Rev Drug Discov. 2004, SupplS3-40

16 X. Wang; Z. Y. Zhang; S. Arora; L. Hughes; J. Wang; D. Powers; J. Christensen; S. Lu; V. Kansra. Effects of Rolapitant Administered Intravenously or Orally on the Pharmacokinetics of Digoxin (P-glycoprotein Substrate) and Sulfasalazine (Breast Cancer Resistance Protein Substrate) in Healthy Volunteers. J Clin Pharmacol. 2018, 58(2): 202-11

17 B. Hughes. 2009 FDA drug approvals. Nat Rev Drug Discov. 2010, 9(2): 89-92

18 E. Germovsek; C. I. Barker; M. Sharland. What do I need to know about aminoglycoside antibiotics? Arch Dis Child Educ Pract Ed. 2017, 102(2): 89-93

19 S. N. Ngo; T. Barnes. Is there variability in drug release and physical characteristics of amiodarone chloride from different commercially available tablets? Possible therapeutic implications. Int J Pharm Pract. 2010, 18(4): 245-8

20 X. W. Chen; E. S. Serag; K. B. Sneed; J. Liang; H. Chew; S. Y. Pan; S. F. Zhou. Clinical herbal interactions with conventional drugs: from molecules to maladies. Curr Med Chem. 2011, 18(31): 4836-50

21 C. J. Kapelios; G. Karamanakos; S. Liatis; M. Sarafadi; M. Polizois; I. Papoutsis; A. D. Kokkinos. Recurrent episodes of life-threatening vasodilatory shock following unintentional intoxication with amlodipine. Hellenic J Cardiol. 2017, 58(5): 369-71

22 S. Harmsen; A. C. McLaren; C. Pauken; R. McLemore. Amphotericin B is cytotoxic at locally delivered concentrations. Clin Orthop Relat Res. 2011, 469(11): 3016-21

23 M. Bretagne; P. Boudou-Rouquette; O. Huillard; A. Thomas-Schoemann; A. Chahwakilian; G. Orvoen; J. Arrondeau; C. Tlemsani; A. Cessot; L. Cabanes; B. Blanchet; R. Coriat; J. Alexandre; F. Goldwasser. [Tyrosine kinase inhibiting the VEGF pathway and elderly people: Tolerance, pre-treatment assessment and side effects management]. Bull Cancer. 2016, 103(3): 259-72

24 M. A. Buhl; G. Gomez; M. V. Collado; E. M. Oddo; M. Khoury; P. J. Azurmendi; J. Sarano. [Genetic polymorphisms of thiopurine methyltransferase and incidence of adverse events in patients with medical indication of azathioprine]. Medicina. 2018, 78(2): 65-70

25 E. Augier; R. S. Dulman; R. Damadzic; A. Pilling; J. P. Hamilton; M. Heilig. The GABAB Positive Allosteric Modulator ADX71441 Attenuates Alcohol Self-Administration and Relapse to Alcohol Seeking in Rats. Neuropsychopharmacology. 2017, 42(9): 1789-99

26 Y. S. Lin; S. J. Kerr; T. Randolph; L. M. Shireman; T. Senn; J. S. McCune. Prediction of Intravenous Busulfan Clearance by Endogenous Plasma Biomarkers Using Global Pharmacometabolomics. Metabolomics. 2016, 12(10):

27 R. Ciftci; F. Tas; S. Karabulut; S. Ciftci. Combination of capecitabine and phenytoin may cause phenytoin intoxication: a case report. Am J Ther. 2015, 22(1): e17-9

28 P. Chandrasekaran; R. Kandasamy. Development of Extended-Release Oral Flexible Tablet (ER-OFT) Formulation for Pediatric and Geriatric Compliance: an Age-Appropriate Formulation. AAPS PharmSciTech. 2017, 18(7): 2394-409

29 E. Perucca. Clinical pharmacology and therapeutic use of the new antiepileptic drugs. Fundam Clin Pharmacol. 2001, 15(6): 405-17

30 A. Rousseau; P. Marquet; J. Debord; C. Sabot; G. Lachatre. Adaptive control methods for the dose individualisation of anticancer agents. Clin Pharmacokinet. 2000, 38(4): 315-53

31 T. M. Davis; D. A. Syed; K. F. Ilett; P. H. Barrett. Toxicity related to chloroquine treatment of resistant vivax malaria. Ann Pharmacother. 2003, 37(4): 526-9

32 I. Taskintuna; F. M. Rahhal; J. F. Arevalo; D. Munguia; A. S. Banker; E. De Clercq; W. R. Freeman. Low-dose intravitreal cidofovir (HPMPC) therapy of cytomegalovirus retinitis in patients with acquired immune deficiency syndrome. Ophthalmology. 1997, 104(6): 1049-57

33 J. Alderman. Coadministration of sertraline with cisapride or pimozide: an open-label, nonrandomized examination of pharmacokinetics and corrected QT intervals in healthy adult volunteers. Clin Ther. 2005, 27(7): 1050-63

34 J. M. Onoda; K. K. Nelson; J. D. Taylor; K. V. Honn. Cisplatin and nifedipine: synergistic antitumor effects against an inherently cisplatin-resistant tumor. Cancer Lett. 1988, 40(1): 39-47

35 P. G. Official-Website. Generic Substitution Law and Narrow Therapeutic Index (NTI) drugs. Pennsylvania Generic Substitution Law. 2015, 1(1): 1-1

36 M. J. Romano; A. Dinh. A 1000-fold overdose of clonidine caused by a compounding error in a 5-year-old child with attention-deficit/hyperactivity disorder. Pediatrics. 2001, 108(2): 471-2

37 C. Hiemke. Consensus Guideline Based Therapeutic Drug Monitoring (TDM) in Psychiatry and Neurology. Curr Drug Deliv. 2016, 13(3): 353-61

38 B. R. Overholser; D. R. Foster. Opioid pharmacokinetic drug-drug interactions. Am J Manag Care. 2011, 17 Suppl 11S276-87

39 Y. Finkelstein; S. E. Aks; J. R. Hutson; D. N. Juurlink; P. Nguyen; G. Dubnov-Raz; U. Pollak; G. Koren; Y. Bentur. Colchicine poisoning: the dark side of an ancient drug. Clin Toxicol. 2010, 48(5): 407-14

40 S. Medani; C. Wall. Colchicine toxicity in renal patients - Are we paying attention? Clin Nephrol. 2016, 86(2): 100-5

41 J. M. Pogue; J. K. Ortwine; K. S. Kaye. Clinical considerations for optimal use of the polymyxins: A focus on agent selection and dosing. Clin Microbiol Infect. 2017, 23(4): 229-33

42 C. Ekhart; A. Gebretensae; H. Rosing; S. Rodenhuis; J. H. Beijnen; A. D. Huitema. Simultaneous quantification of cyclophosphamide and its active metabolite 4-hydroxycyclophosphamide in human plasma by high-performance liquid chromatography coupled with electrospray ionization tandem mass spectrometry (LC-MS/MS). J Chromatogr B Analyt Technol Biomed Life Sci. 2007, 854(1-2): 345-9

43 K. A. Rodvold. Clinical pharmacokinetics of clarithromycin. Clin Pharmacokinet. 1999, 37(5): 385-98

44 D. S. Official-Website. Dalteparin Sodium Drug Summary. Perscribers' Digital Reference. 2018, 1(1): 1-1

45 E. Gozalpour; R. Greupink; A. Bilos; V. Verweij; J. J. van den Heuvel; R. Masereeuw; F. G. Russel; J. B. Koenderink. Convallatoxin: a new P-glycoprotein substrate. Eur J Pharmacol. 2014, 74418-27

46 J. D. Henderson; R. H. Esham. Generic substitution: issues for problematic drugs. South Med J. 2001, 94(1): 16-21

47 R. Valdes, Jr.; S. A. Jortani; M. Gheorghiade. Standards of laboratory practice: cardiac drug monitoring. National Academy of Clinical Biochemistry. Clin Chem. 1998, 44(5): 1096-109

48 K. Eckmann; L. B. Michaud; E. Rivera; T. L. Madden; L. Esparza-Guerra; J. Kawedia; D. J. Booser; M. C. Green; G. N. Hortobagyi; V. Valero. Pilot study to assess toxicity and pharmacokinetics of docetaxel in patients with metastatic breast cancer and impaired liver function secondary to hepatic metastases. J Oncol Pharm Pract. 2014, 20(2): 120-9

49 Y. L. Han; H. L. Yu; D. Li; X. L. Meng; Z. Y. Zhou; Q. Yu; X. Y. Zhang; F. J. Wang; C. Guo. Inhibitory effects of limonin on six human cytochrome P450 enzymes and P-glycoprotein in vitro. Toxicol In Vitro. 2011, 25(8): 1828-33

50 P. Naidoo; V. V. Chetty; M. Chetty. Impact of CYP polymorphisms, ethnicity and sex differences in metabolism on dosing strategies: the case of efavirenz. Eur J Clin Pharmacol. 2014, 70(4): 379-89

51 F. E. Simons. Epinephrine (adrenaline) in the first-aid, out-of-hospital treatment of anaphylaxis. Novartis Found Symp. 2004, 257228-43; discussion 43-7, 76-85

52 R. J. Cersosimo; W. K. Hong. Epirubicin: a review of the pharmacology, clinical activity, and adverse effects of an adriamycin analogue. J Clin Oncol. 1986, 4(3): 425-39

53 I. A. Najar; R. K. Johri. Pharmaceutical and pharmacological approaches for bioavailability enhancement of etoposide. J Biosci. 2014, 39(1): 139-44

54 T. van Gelder; L. Fischer; F. Shihab; M. Shipkova. Optimizing everolimus exposure when combined with calcineurin inhibitors in solid organ transplantation. Transplant Rev. 2017, 31(3): 151-57

55 D. Padhi; R. Harris. Clinical pharmacokinetic and pharmacodynamic profile of cinacalcet hydrochloride. Clin Pharmacokinet. 2009, 48(5): 303-11

56 M. W. Saif; R. B. Diasio. Benefit of uridine triacetate (Vistogard) in rescuing severe 5-fluorouracil toxicity in patients with dihydropyrimidine dehydrogenase (DPYD) deficiency. Cancer Chemother Pharmacol. 2016, 78(1): 151-6

57 J. Fareed; D. A. Hoppensteadt; R. L. Bick. Management of thrombotic and cardiovascular disorders in the new millenium. Clin Appl Thromb Hemost. 2003, 9(2): 101-8

58 O. K. Thu; O. Spigset; O. G. Nilsen; B. Hellum. Effect of commercial Rhodiola rosea on CYP enzyme activity in humans. Eur J Clin Pharmacol. 2016, 72(3): 295-300

59 C. Thirukkumaran; D. G. Morris. Oncolytic Viral Therapy Using Reovirus. Methods Mol Biol. 2015, 1317187-223

60 C. Mercier; C. Raynal; L. Dahan; A. Ortiz; A. Evrard; C. Dupuis; A. Blesius; M. Duluc; F. Franceschini; S. Giacometti; S. Salas; G. Milano; R. Favre; J. F. Seitz; J. Ciccolini. Toxic death case in a patient undergoing gemcitabine-based chemotherapy in relation with cytidine deaminase downregulation. Pharmacogenet Genomics. 2007, 17(10): 841-4

61 K. Brooks. Anticoagulation Drugs: What Nurses Need to Know. Johns Hopkins Nursing. 2011, 7(1): 1-1

62 J. Ward; N. Lewis; D. A. Tsitsikas. Improving routine outpatient monitoring for patients with sickle-cell disease on hydroxyurea. BMJ Open Qual. 2018, 7(1): e000218

63 S. H. Preskorn. Inadvertent fatal imipramine poisoning of a child: what happened to Tommy? J Psychiatr Pract. 2011, 17(2): 118-23

64 N. C. Kaarsholm; S. Lin; L. Yan; T. Kelly; M. van Heek; J. Mu; M. Wu; G. Dai; Y. Cui; Y. Zhu; E. Carballo-Jane; V. Reddy; P. Zafian; P. Huo; S. Shi; V. Antochshuk; A. Ogawa; F. Liu; S. C. Souza; W. Seghezzi; J. L. Duffy; M. Erion; R. P. Nargund; D. E. Kelley. Engineering Glucose Responsiveness Into Insulin. Diabetes. 2018, 67(2): 299-308

65 S. L. Pogue; T. Taura; M. Bi; Y. Yun; A. Sho; G. Mikesell; C. Behrens; M. Sokolovsky; H. Hallak; M. Rosenstock; E. Sanchez; H. Chen; J. Berenson; A. Doyle; S. Nock; D. S. Wilson. Targeting Attenuated Interferon-alpha to Myeloma Cells with a CD38 Antibody Induces Potent Tumor Regression with Reduced Off-Target Activity. PLoS One. 2016, 11(9): e0162472

66 K. L. Mealey; S. Dassanayake; N. S. Burke. Establishment of a cell line for assessing drugs as canine P-glycoprotein substrates: proof of principle. J Vet Pharmacol Ther. 2017, 40(5): 545-51

67 G. E. Schumacher. Using pharmacokinetics in drug therapy. VII: Pharmacokinetic factors influencing drug therapy in the aged. Am J Hosp Pharm. 1980, 37(4): 559-62

68 C. Gokbulut; H. S. Yalinkilinc; D. Aksit; V. Veneziano. Comparative pharmacokinetics of levamisole-oxyclozanide combination in sheep and goats following per os administration. Can J Vet Res. 2014, 78(4): 316-20

69 B. M. Parker; B. J. Cusack; R. E. Vestal. Pharmacokinetic optimisation of drug therapy in elderly patients. Drugs Aging. 1995, 7(1): 10-8

70 K. Nolan; J. Kamrath; J. Levitt. Lindane toxicity: a comprehensive review of the medical literature. Pediatr Dermatol. 2012, 29(2): 141-6

71 S. Wasserman; G. Meintjes; G. Maartens. Linezolid in the treatment of drug-resistant tuberculosis: the challenge of its narrow therapeutic index. Expert Rev Anti Infect Ther. 2016, 14(10): 901-15

72 A. J. Kansagra; E. Yang; S. Nambiar; P. S. Patel; M. S. Karetzky. A rare case of acute respiratory distress syndrome secondary to acute lithium intoxication. Am J Ther. 2014, 21(2): e31-4

73 M. Weiser; S. Burshtein; A. A. Gershon; G. Marian; N. Vlad; I. G. Grecu; E. Tocari; A. Tiugan; M. Hotineanu; J. M. Davis. Allopurinol for mania: a randomized trial of allopurinol versus placebo as add-on treatment to mood stabilizers and/or antipsychotic agents in manic patients with bipolar disorder. Bipolar Disord. 2014, 16(4): 441-7

74 S. Sahasranaman; D. Howard; S. Roy. Clinical pharmacology and pharmacogenetics of thiopurines. Eur J Clin Pharmacol. 2008, 64(8): 753-67

75 S. Ouwerkerk-Mahadevan; J. Snoeys; M. Peeters; M. Beumont-Mauviel; A. Simion. Drug-Drug Interactions with the NS3/4A Protease Inhibitor Simeprevir. Clin Pharmacokinet. 2016, 55(2): 197-208

76 I. Meineke; H. Steinmetz; J. Kirchheiner; J. Brockmoller. Therapeutic drug monitoring of mirtazapine, desmethylmirtazapine, 8-hydroxymirtazapine, and mirtazapine-N-oxide by enantioselective HPLC with fluorescence detection. Ther Drug Monit. 2006, 28(6): 760-5

77 I. A. Shah; W. E. Lindup; P. G. McCulloch. Variability of mitomycin C adsorption by activated charcoal. J Pharm Pharmacol. 1998, 50(3): 251-6

78 D. Theile; W. E. Haefeli; J. Weiss. Effects of adrenolytic mitotane on drug elimination pathways assessed in vitro. Endocrine. 2015, 49(3): 842-53

79 L. M. Shaw; D. W. Holt; P. Keown; R. Venkataramanan; R. W. Yatscoff. Current opinions on therapeutic drug monitoring of immunosuppressive drugs. Clin Ther. 1999, 21(10): 1632-52; discussion 31

80 C. Mendelsohn. Managing nicotine dependence in NSW hospitals under the Smoke-free Health Care Policy. Public Health Res Pract. 2015, 25(3): e2531533

81 P. Gjerden; L. Slordal. [Clinical pharmacology of anticholinergic antiparkinson agents. A review with emphasis on acute toxicity]. Tidsskr Nor Laegeforen. 1998, 118(1): 53-5

82 S. J. Hwang; J. W. Park; S. D. Lee; G. J. Kim; C. H. Sin; S. H. Nam; B. S. Kim. Capecitabine and oxaliplatin (XELOX) for the treatment of patients with metastatic gastric cancer and severe liver dysfunction. Korean J Intern Med. 2006, 21(4): 252-5

83 C. A. Grotegut; E. Ngan; M. E. Garrett; M. L. Miranda; A. E. Ashley-Koch; G. K. Swamy. The association of single-nucleotide polymorphisms in the oxytocin receptor and G protein-coupled receptor kinase 6 (GRK6) genes with oxytocin dosing requirements and labor outcomes. Am J Obstet Gynecol. 2017, 217(3): 367 e1-67 e9

84 S. Minhas; N. Setia; S. Pandita; R. Saxena; I. C. Verma; S. Aggarwal. Prevalence of CYP2C8 polymorphisms in a North Indian population. Genet Mol Res. 2013, 12(3): 2260-6

85 M. M. Posada; J. A. Bacon; K. B. Schneck; R. G. Tirona; R. B. Kim; J. W. Higgins; Y. A. Pak; S. D. Hall; K. M. Hillgren. Prediction of renal transporter mediated drug-drug interactions for pemetrexed using physiologically based pharmacokinetic modeling. Drug Metab Dispos. 2015, 43(3): 325-34

86 S. M. Coupey. Barbiturates. Pediatr Rev. 1997, 18(8): 260-4; quiz 65

87 M. H. Umer Usman; S. Raza; S. Raza; M. Ezekowitz. Advancement in antithrombotics for stroke prevention in atrial fibrillation. J Interv Card Electrophysiol. 2008, 22(2): 129-37

88 S. Barbour; T. Smit; X. Wang; D. Powers; S. Arora; V. Kansra; M. Aapro; J. Herrstedt. Integrated safety analysis of rolapitant with coadministered drugs from phase II/III trials: an assessment of CYP2D6 or BCRP inhibition by rolapitant. Ann Oncol. 2017, 28(6): 1268-73

89 M. A. Kuhn. Herbal remedies: drug-herb interactions. Crit Care Nurse. 2002, 22(2): 22-8, 30, 32; quiz 34-5

90 A. Dhir. Propofol in the treatment of refractory migraine headaches. Expert Rev Neurother. 2016, 16(9): 1007-11

91 C. Becker; J. B. Dressman; G. L. Amidon; H. E. Junginger; S. Kopp; K. K. Midha; V. P. Shah; S. Stavchansky; D. M. Barends. Biowaiver monographs for immediate release solid oral dosage forms: pyrazinamide. J Pharm Sci. 2008, 97(9): 3709-20

92 D. Soltanifar; B. Carvalho; P. Sultan. Perioperative considerations of the patient with malaria. Can J Anaesth. 2015, 62(3): 304-18

93 N. Goodarzi; A. Barazesh Morgani; B. Abrahamsson; R. Cristofoletti; D. W. Groot; P. Langguth; M. U. Mehta; J. E. Polli; V. P. Shah; J. B. Dressman. Biowaiver Monographs for Immediate Release Solid Oral Dosage Forms: Ribavirin. J Pharm Sci. 2016, 105(4): 1362-9

94 B. Golubovic; M. Prostran; B. Miljkovic; K. Vucicevic; D. Radivojevic; I. Grabnar. Population Pharmacokinetic Approach of Immunosuppressive Therapy in Kidney Transplant Patients. Curr Med Chem. 2016, 23(19): 1998-2011

95 R. M. Velickovic-Radovanovic; G. Paunovic; M. Mikov; V. Djordjevic; M. Stojanovic; A. Catic-Djordjevic; T. Cvetkovic. Clinical pharmacokinetics of tacrolimus after the first oral administration in renal transplant recipients on triple immunosuppressive therapy. Basic Clin Pharmacol Toxicol. 2010, 106(6): 505-10

96 J. A. Ohar; J. F. Donohue. Mono- and combination therapy of long-acting bronchodilators and inhaled corticosteroids in advanced COPD. Semin Respir Crit Care Med. 2010, 31(3): 321-33

97 H. R. Henney, 3rd; A. Fitzpatrick; J. Stewart; J. D. Runyan. Relative bioavailability of tizanidine hydrochloride capsule formulation compared with capsule contents administered in applesauce: a single-dose, open-label, randomized, two-way, crossover study in fasted healthy adult subjects. Clin Ther. 2008, 30(12): 2263-71

98 V. Madsen; A. Lind; M. Rasmussen; K. Coulthard. Determination of tobramycin in saliva is not suitable for therapeutic drug monitoring of patients with cystic fibrosis. J Cyst Fibros. 2004, 3(4): 249-51

99 J. O. Miners; D. J. Birkett. Cytochrome P4502C9: an enzyme of major importance in human drug metabolism. Br J Clin Pharmacol. 1998, 45(6): 525-38

100 C. S. Bouman; H. J. van Kan; R. P. Koopmans; J. C. Korevaar; M. J. Schultz; M. B. Vroom. Discrepancies between observed and predicted continuous venovenous hemofiltration removal of antimicrobial agents in critically ill patients and the effects on dosing. Intensive Care Med. 2006, 32(12): 2013-9

101 R. Said; A. M. Tsimberidou. Pharmacokinetic evaluation of vincristine for the treatment of lymphoid malignancies. Expert Opin Drug Metab Toxicol. 2014, 10(3): 483-94

102 N. K. Ibrahim; V. Valero; Z. Rahman; R. L. Theriault; R. S. Walters; A. U. Buzdar; D. J. Booser; F. A. Holmes; J. L. Murray, 3rd; J. Willey; R. Bast; G. N. Hortobagyi. Phase I-II vinorelbine (Navelbine) by continuous infusion in patients with metastatic breast cancer: cumulative toxicities limit dose escalation. Cancer Invest. 2001, 19(5): 459-66

103 R. S. Kadam; J. N. Van Den Anker. Pediatric Clinical Pharmacology of Voriconazole: Role of Pharmacokinetic/Pharmacodynamic Modeling in Pharmacotherapy. Clin Pharmacokinet. 2016, 55(9): 1031-43
